# A cross platform m7G risk score based on five gene pairs stratifies prognosis and stemness in acute myeloid leukemia

**DOI:** 10.1007/s12672-026-05464-5

**Published:** 2026-06-14

**Authors:** Ruolin Guo, Zeyu Deng

**Affiliations:** 1 Hunan Prevention and Treatment Institute for Occupational Diseases, Changsha, Hunan P. R. China; 2 Department of Hematology, The Second Xiangya Hospital of Central South University, Changsha, Hunan P. R. China

**Keywords:** M7G, LSC, AML, Bioinformatics

## Abstract

AML exhibits marked heterogeneity in genetics, stemness, and treatment response. We profiled m7G regulator expression across multi-cohort AML transcriptomes and identified two m7G-related phenotypes with distinct survival and enrichment of adverse cytogenetics and primitive hierarchy. We then developed a cross-platform five–gene-pair m7G risk score using LASSO-Cox modeling. The score robustly stratified overall survival in TCGA and external cohorts (BEAT AML and GSE37642) and remained an independent prognostic factor. High risk was associated with increased leukemic stemness, including enrichment of HSC/MPP-like states in single-cell AML and higher scores in LSC+ fractions. The score also discriminated induction response (AUC 0.838) and increased in paired diagnosis-relapse samples, supporting relevance to chemoresistance. Drug sensitivity integration further nominated candidate compounds with preferential activity in high-risk AML. These findings connect m7G-related transcriptional programs to AML risk and provide a practical tool for refined prognostication and therapeutic prioritization.

**Supplementary Information:**

The online version contains supplementary material available at 10.1007/s12672-026-05464-5.

## Introduction

Acute Myeloid Leukemia (AML) manifests as a hematological malignancy with pronounced patient-specific heterogeneity. This heterogeneity is driven by a broad array of genomic alterations and a multitude of molecular mutation profiles, thereby necessitating a diversified approach to AML therapeutics [1]. The 2017 European LeukemiaNet (ELN) guidelines stratify AML patients into three prognostic categories—favorable, intermediate, and adverse—integrating an assortment of cellular karyotypes and gene mutations [2]. Nonetheless, a subset of patients with ostensibly normal karyotypes faces a grim prognosis characterized by resistant and recurrent disease, suggesting that variations in leukemic stem cell (LSC) stemness may underlie another dimension of AML heterogeneity [3–5]. A risk model named LSC17, devised by Stanley W. K. Ng et al., harnesses the expression of genes upregulated in LSCs [6]. Complementarily, Andy G. X. Zeng and colleagues have developed a cellular hierarchy model that synergizes genetic data and stem cell dynamics in AML, delineating patients into four cellular states: primitive, granulocyte-macrophage progenitor (GMP)-like, intermediate, and mature [7]. Despite advancements, conventional chemotherapy remains the cornerstone of AML treatment, with post-therapy relapse and induction failure contributing significantly to therapeutic adversity [8]. The advent of novel treatment modalities has not eclipsed the inherent treatment response heterogeneity, underscoring the imperative for nuanced exploration of AML heterogeneity to refine personalized treatment paradigms.

The N7-methylguanosine (m7G) modification, ubiquitous across RNA species, emerges as a key post-transcriptional modification within the 5’ caps of eukaryotic mRNAs and is extensively involved in the regulatory architecture of rRNA and tRNA [9–11]. Recent discoveries have shed light on a cadre of m7G regulatory genes. Specifically, METTL1 and WDR4 have been implicated in the m7G modification of tRNA, miRNA, and mRNA [12–15], while RNMT and RAM orchestrate m7G modifications at the mRNA 5’ caps [16, 17]. WBSCR22 and TRMT11 play pivotal roles in the m7G modification processes within rRNA [18, 19]. The RNA-binding protein QKI has been identified as an interactor with m7G sites, enhancing mRNA stability and translational efficiency [20]. The first six m7G regulatory proteins—METTL1, WDR4, RNMT, RAM, WBSCR22, and TRMT11—function as m7G writers, whereas QKI serves as an m7G reader. Growing evidence indicates that m7G plays a critical role in tumorigenesis and progression by modulating the expression of oncogenes and tumor suppressor genes [21]. Numerous studies have demonstrated that m7G is involved in the pathogenesis and drug resistance of AML. Notably, elevated expression of METTL1 and WDR4 has been observed at both mRNA and protein levels in AML patient samples. Targeted suppression of METTL1 markedly attenuates the proliferation of LSCs with minimal collateral effects on normal hematopoietic progenitors. In vivo, METTL1-deficient mice demonstrate reduced tumor burden and extended survival [22]. Furthermore, m7G has been implicated in the pathogenesis and chemoresistance of AML, with differential m7G modification profiles identified in resistant AML cell phenotypes, including alterations in messenger RNA, long non-coding RNA, and circular RNA [23–25]. Such evidence solidifies the connection between m7G dynamics and AML progression and drug resistance. Prior studies, however, have been constrained to cell lines and animal models, and thus may not fully capture the complexity of AML heterogeneity. Our study aims to identify specific m7G regulatory patterns in the AML population by analyzing a large array of AML patient samples. Utilizing transcriptome data of previously identified m7G regulatory genes, we categorized AML samples into distinct groups and developed a risk scoring model to quantify the risk for each AML patient, thereby identifying high-risk groups. Subsequently, we examined the immune microenvironment, genomic variations, leukemia stemness, and drug response differences among samples with varying risk stratifications. In summary, our research seeks to elucidate the potential links between heterogeneity, pathogenic mechanisms, drug resistance, and m7G in AML patients from an array-based perspective, offering new strategies for the precision treatment of AML.

## Methods

### Data collection and processing

The seven m7G-related genes analyzed in this study, including METTL1, WDR4, RNMT, BUD23, TRMT112, QKI, and RAB27A, were selected a priori based on prior functional annotation as m7G regulators or m7G-associated regulatory factors. These genes cover major m7G-related regulatory layers and were consistently detectable across the AML transcriptomic cohorts used for cross-platform analysis. The acquisition and processing of gene expression profiles, along with corresponding clinical data for newly diagnosed Acute Myeloid Leukemia (AML) samples, was meticulously carried out from an array of eminent sources. These included The Cancer Genome Atlas (TCGA) [26], the BEAT AML initiative [27], and the Gene Expression Omnibus (GEO) repository. RNA-Seq data from TCGA and BEAT were scrupulously identified, whereas the GSE37642 dataset was constituted of comprehensive microarray data.

Uniformly processed single-cell RNA-seq data for pediatric acute myeloid leukemia (AML) were obtained from the Single-cell Pediatric Cancer Atlas (ScPCA) Portal (project SCPCP000007). The dataset comprised 26 AML samples generated using the 10x Genomics Chromium 5′ v2 chemistry (10xv2_5prime). Downstream analyses were performed using the Seurat R package [28]. Cells were retained if they satisfied the following quality-control criteria: mitochondrial transcript fraction (percent.mt) < 15% and the number of detected genes (nFeature_RNA) > 200, resulting in a final dataset of 140,653 cells. Cell-type annotation was performed using ‘BoneMarrowMap’ with its two-stage reference-mapping strategy [29]. For each AML sample, a pseudo-bulk expression matrix was constructed and normalized to counts per million (CPM). An m7G risk score was then calculated for each sample using a five–gene-pair scoring formula; samples were dichotomized into high- and low-risk groups based on the cohort median risk score. Differences in cell-type proportions between the two risk groups were visualized using boxplots.

Additionally, microarray gene expression profiling was meticulously conducted on 138 LSC + and 89 LSC– cell fractions, delineated in dataset GSE76008 [6]. The calculation method for the LSC17 risk score is based on the research findings of Stanley W.K. Ng et al. [6].We acquired transcriptomic data from the GSE103424 dataset, which includes initial diagnosis AML samples with treatment response information: 18 samples with induction failure and 18 samples with complete remission. Additionally, the transcriptomic data for initial diagnosis-relapse paired AML samples were obtained from Christopher et al. [30]. (9 initial diagnosis-relapse pairs) and the GSE83533 dataset (19 initial diagnosis-relapse pairs) (Supplementary Table 1). Standardization of RNA-Seq gene expression data employed TPM or RPKM normalization techniques, ensuring robust comparability, while microarray data normalization was adeptly conducted using the `normalizeBetweenArrays` function within the ‘limma’ package [31]. All gene expression values underwent a logarithmic transformation to enhance the analytical fidelity of the dataset.

### Unsupervised clustering approach

We utilized the ‘ConsensusClusterPlus’ package [32] in R for unsupervised clustering of AML samples, employing the k-means algorithm with Euclidean distance as the metric for sample comparison.

### m7G signature formulation

Using the ‘limma’ package, we identified differentially expressed genes between cluster A and cluster B, selecting 1,867 genes that met the significance threshold of FDR < 0.05 and |logFC| > 0.5 for further analysis. Considering the availability of genes in validation arrays, we excluded those not present in BEAT or GSE37642 datasets, resulting in a final set of 1,017 genes. Cox regression analysis was performed on 1,017 genes, and prognostic genes with p < 0.01 were selected, resulting in 82 genes. These genes were used to generate 82*81/2 gene pairs. A gene pair was assigned a value of 1 if gene A’s expression was higher than gene B’s, and − 1 otherwise. Gene pairs consistently showing values of 1 or -1 in more than 80% or less than 20% of samples were discarded due to their variability. The remaining gene pairs were subjected to univariate Cox analysis, retaining those with p < 0.01, which resulted in 180 gene pairs for the construction of the m7G signature. A multifactorial Cox Lasso regression analysis was utilized to construct the m7G prognostic model, with the Lasso technique compressing the Cox coefficients of gene pairs less associated with prognosis, ultimately forming a signature of five gene pairs (Supplementary Fig. 1). The risk score was calculated as follows: RiskScore = Σ(p * c), where ‘p’ represents the gene pair value (1 or -1), and ‘c’ is the regression coefficient. Owing to the model’s robustness across various platforms and data batches, it demonstrates strong clinical utility. The median risk score from the training set AML samples was used as the cutoff to stratify both the training and validation sets into high and low-risk groups.

### Statistical analysis framework

This study harnessed the power of R software (version 4.3.0) for all computational analyses. SNP interrogation was adeptly managed using the ‘maftools’ package [33].The AUC values were calculated with the precision of the ‘pROC’ package [34], and the estimation of the population mean AUC from the sample was conducted using the bootstrap method with 1000 iterations of resampling, matching the original sample size in each. Pathway enrichment analysis was meticulously performed utilizing the ‘clusterProfiler’ package [35]. The construction of Kaplan–Meier overall survival curves and the execution of Cox regression analyses were rendered using the ‘Survival’ and ‘survminer’ packages, respectively. The ‘glmnet’ package [36] facilitated the Lasso Cox regression analysis, providing a refined model selection approach. Survival analysis p-values were computed via the log-rank test, ensuring rigorous assessment of statistical differences. Group comparisons were conducted with the Mann-Whitney U test or Kruskal-Wallis H test, depending on the data structure. The entire suite of visualizations was crafted through the artful use of ‘ggplot2’, with a p-value threshold of less than 0.05 set to demarcate statistical significance.

## Results

### Identification of two m7g subtypes

We initially scrutinized single nucleotide polymorphisms (SNPs) and copy number variations (CNVs) of these seven m7G regulatory genes. Based on data from The Cancer Genome Atlas (TCGA), only QKI exhibited a modest somatic mutation rate (1.18%), with no SNPs detected in the remaining genes (Supplementary Fig. 2A). The incidence of CNVs in the seven m7G genes within AML samples was not pronounced; however, BUD23, RAB27A and RNMT displayed significant heterozygous copy number loss (7.85%,2.09% and 3.14%, respectively), while TRMT112 and WDR4 demonstrated copy number amplification (3.14% and 6.81%, respectively) (Supplementary Fig. 2B). Correlative analysis revealed no significant association between the CNVs of the seven m7G genes and their mRNA expression levels (Supplementary Fig. 2C). Nevertheless, copy number variations of two genes were correlated with overall survival in AML; WDR4’s copy number gain was indicative of a worse prognosis (Supplementary Fig. 2D), while RNMT’s copy number loss suggested an adverse outcome (Supplementary Fig. 2E). Moreover, higher expression of WDR4 was significantly associated with decreased survival, and lower expression of RNMT was linked to poorer prognosis. Additionally, Kaplan–Meier survival analyses in the TCGA cohort indicated that patients with higher expression of RAB27A and RNMT experienced improved survival, whereas higher expression of METTL1, TRMT112, and WDR4 was associated with worse survival. QKI expression showed no significant association with survival (Supplementary Fig. 2F–K).

**Fig. 1:**
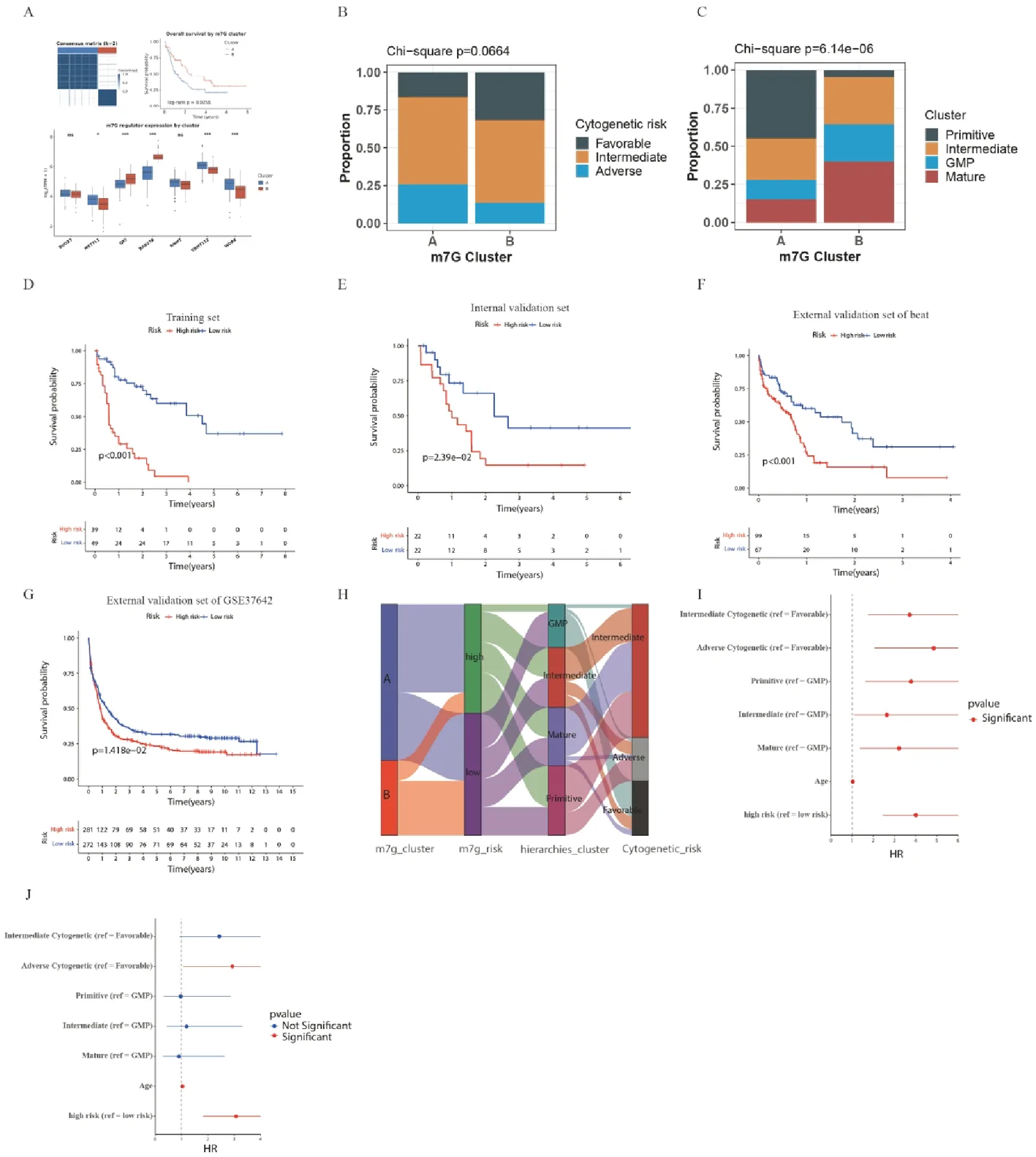
Identification of two distinct m7g molecular clusters and formulation of the m7g Signature. **A** Utilizing consensus clustering predicated on the expression profiles of seven pivotal m7g regulatory genes, two disparate m7g subtypes were discerned. Box plots delineate the differential expression of these seven m7g genes across the identified clusters. Kaplan-Meier survival analyses elucidate disparities in overall survival among AML patients characterized by these m7g subtypes. **B** The distribution of three cytogenetic classifications within the m7g subgroups is quantified. **C** Additionally, the proportion of four cellular hierarchy levels across the m7g subgroups is evaluated. **D**–**G** Kaplan-Meier plots underscore a significant prognostic decrement in overall survival for AML patients within the m7g high-risk category, compared to their low-risk counterparts, across training, internal validation, and two external validation cohorts. **H** A Sankey diagram illustrates the interrelationships among the m7g clusters, risk stratification, cellular hierarchies, and cytogenetic categories. **I**–**J** Results from univariate and multivariate Cox regression analyses are presented, integrating the m7g risk model with other clinical parameters

In our study, unsupervised clustering of AML patients from the TCGA database, based on expression data from seven m7G regulatory genes, yielded two distinct clusters, designated A and B (Fig. 1A; Supplementary Table 2). Differential expression analysis identified marked overexpression of METTL1, WDR4, and TRMT112 in cluster A, with QKI and RAB27A significantly upregulated in cluster B (Fig. 1A). The Kaplan-Meier survival curve highlighted a notably poorer prognosis for patients in cluster A (*p* = 0.026) (Fig. 1A). Karyotypic analyses between the two clusters revealed a significantly higher proportion of adverse cytogenetics in cluster A (26% vs. 14%) (Fig. 1B). Furthermore, an evaluation of AML cell hierarchies within these clusters unveiled a significant predominance of Primitive type in cluster A (45% compared to 4% in cluster B), indicative of a more pronounced stemness in AML leukemia stem cells within cluster A (Fig. 1C). In summary, our findings cast light on the intricate interplay between m7G modification patterns, genomic variations, and the hierarchy of leukemia stem cells in AML.

### Development and evaluation of an m7G-associated signature

Previously, we identified two m7G-related subtypes of AML (Group A and Group B) by unsupervised clustering; however, we did not directly quantify m7G modification levels in individual samples. To enable sample-level quantification with clinical applicability, we developed an m7G-associated prognostic signature using differentially expressed genes between Group A and Group B. To improve robustness across platforms, we adopted a gene-pair comparison strategy and applied LASSO-penalized Cox regression to remove weak predictors, yielding a final model comprising five gene pairs: ETS2|AGTPBP1, LY6E|AGTPBP1, PNMA1|DGKG, ACOT7|STAR, and IL2RA|TCF4 (Supplementary Fig. 3A; Supplementary Table 3). For model development, two-thirds of TCGA-LAML samples were randomly assigned to the training set and the remaining one-third to internal validation; BEAT AML and GSE37642 were used as external validation cohorts. Gene-pair indicator definition: Pair(G1|G2) = 1 if Expr(G1) > Expr(G2); otherwise Pair(G1|G2) = -1.Explicit m7G risk score equation (with coefficients): RiskScore = 0.032510875 * Pair(ETS2|AGTPBP1) + 0.025408167 * Pair(LY6E|AGTPBP1) + 0.008150066 * Pair(PNMA1|DGKG) + 0.088023146 * Pair(ACOT7|STAR) + 0.204135485 * Pair(IL2RA|TCF4).

We calculated RiskScore for each AML sample and used the median score from the TCGA training set (-0.226089523) as the fixed cut-off to stratify samples into high-risk (RiskScore >= -0.226089523) and low-risk (RiskScore < -0.226089523) groups (Supplementary Tables 4–7). Kaplan-Meier analyses consistently showed significantly shorter overall survival in the high-risk group across the training set, the internal validation set, and the external validation cohorts (Fig. 1D-G).

To substantiate the m7G risk score’s capacity for stratifying m7G subtypes in Acute Myeloid Leukemia (AML), Sankey diagrams further corroborated that a majority of high-risk profiles were predominantly derived from Subgroup A (Fig. 1H). The KEGG pathway enrichment analysis for genes overexpressed in Group A and the high-risk echelon delineated 11 pathways significantly enriched, ranging from adaptive immunity to oncogenic PDL1 and PD1 checkpoints (Supplementary Fig. 3B).

### The m7G risk stratification proved to be an independent prognostic factor

To affirm the m7G risk model’s status as an autonomous prognostic indicator, we conducted multivariate Cox regression analyses. This encompassed an array of variables such as age, cytogenetic risk, and leukemia stem cell-like hierarchies, juxtaposed with the m7G prognostic model. The m7G risk score retained its significance as a prognostic determinant in both uni- and multi-factorial Cox analyses, underscoring its independence (Fig. 1I-J).

### Elevated incidence of unfavorable karyotypes in the high-risk cohort

**Fig. 2:**
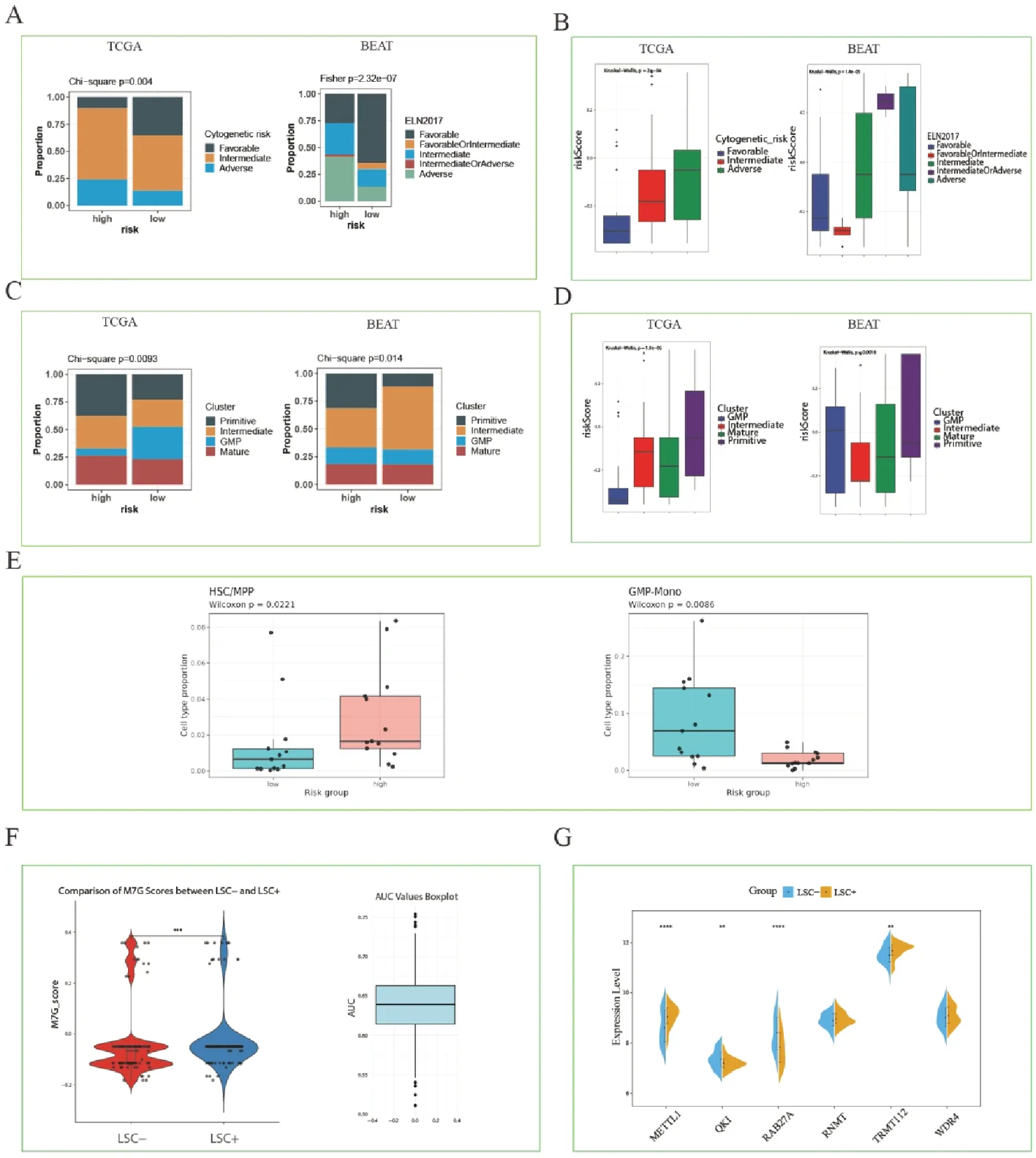
Association Analysis of m7g Risk Scores with features in AML. **A **Proportions of various cytogenetic profiles are compared between high and low m7g risk groups, utilizing datasets from TCGA and BEAT, the latter adopting the ELN2017 classification. **B** Box plots expound on the risk score distributions amongst diverse cytogenetic groups. **C** The distribution of cell hierarchy levels is contrasted between high and low-risk groups within TCGA and BEAT arrays. **D** Box plots elucidate the dispersion of m7g risk scores across different cellular hierarchies. (**E**) shows two boxplots comparing the proportions of three cell types between the high- and low-m7G risk groups, based on analyses of the single-cell RNA-seq dataset. **F** Violin plots compare the distribution of m7G risk scores between leukemia stem cell-negative (LSC−) and leukemia stem cell-positive (LSC+) fractions. The boxplot on the right shows the distribution of AUC values derived from 1,000 bootstrap resamples, with a mean AUC of 0.639. **G** Expression variations of these 6 m7g regulatory genes manifest distinctly between LSC + and LSC- cohorts. A p-value of less than 0.05 denotes statistical significance. Symbols *, **, ***, and **** correspond to p-values of < 0.05, < 0.01, < 0.001, and < 0.0001, respectively

In our comparative analysis of two m7G subtypes, we observed a markedly higher incidence of adverse karyotypes in Group A compared to Group B. Using similar methods, we evaluated the differences in karyotype frequency between high-risk and low-risk cohorts, employing chi-square tests to establish statistical significance. The TCGA high-risk cohort exhibited a significantly increased prevalence of unfavorable karyotypes when compared to the low-risk cohort (24% versus 14%; *p* = 0.004). This finding was mirrored in the analysis of ELN2017 risk stratification within the beat array, which showed a disproportionate representation of adverse karyotypes in the high-risk group (41% versus 13%; *p* < 0.001) (Fig. 2A). Additionally, we assessed the gradation of risk scores across different risk stratifications, noting a consistent decrease from the adverse to intermediate and favorable groups in both TCGA and beat arrays (Fig. 2B), suggesting a strong link between specific m7G risk profiles and cytogenetic aberrations. Hence, the m7G risk model robustly aligns with cytogenetic risk, with high-risk cases showing a consistent enrichment of adverse karyotypes across datasets.

### The m7G pattern is closely related to the stemness of AML

Our preliminary analysis into m7G subgroupings revealed a strikingly higher proportion of primitive cell phenotypes within Group A as compared to Group B. This disparity was also evident upon expanding the comparative framework to encompass cohorts of varying risk profiles, with primitive cell phenotypes being significantly more prevalent within the high-risk cohort—a trend that was consistently evident in analyses of both The Cancer Genome Atlas (TCGA) and the beat array datasets (38% vs. 23% in TCGA, Fig. 2A and 31% vs. 12% in beat, Fig. 2C). Further exploration into the distribution of risk scores across different leukemia cell hierarchies disclosed that primitive cell phenotypes were associated with markedly higher risk scores (Fig. 2D), intimating a potential interconnection between m7G modification patterns and the stemness attribute in acute myeloid leukemia (AML).

In pursuit of a more nuanced understanding, we delved into the expression variations of the m7G regulatory genes across the specified leukemia cell hierarchies. Notably, the expression profiles of this gene sextet demonstrated high concordance in both TCGA and beat arrays (Supplementary Fig. 4). Specifically, METTL1 was significantly downregulated in mature phenotypes, whereas it showed upregulation in intermediate ones. Conversely, QKI expression was predominantly upregulated in mature phenotypes. RAB27A was markedly downregulated in primitive phenotypes, while RNMT, WRD4, and TRMT112 exhibited similar downregulation trends in mature phenotypes. The pronounced differential expression of these genes, in conjunction with previous findings, robustly accentuates the profound nexus between m7G modifications and leukemic cell hierarchies.

To further substantiate the relationship between the m7G risk score and cellular differentiation hierarchy, we analyzed a single-cell RNA-seq dataset comprising 26 AML samples. For each sample, we generated a pseudo-bulk expression matrix (CPM-normalized) and calculated the m7G risk score. Samples were then dichotomized into high- and low-risk groups using the cohort median score. By comparing cell-type composition between the two groups, we observed a significant enrichment of HSC/MPP cells in the high-risk group, whereas GMP–monocyte populations were increased in the low-risk group (Fig. 2E). These findings are consistent with our earlier results, supporting that a higher m7G risk score is associated with a more primitive, stem-like cellular state. In addition, the CD8 + effector memory T-cell subset 2 was significantly enriched in the low-risk group (Supplementary Fig. 3C), suggesting a potential link between the m7G risk score and T-cell–related immune features.

Beyond the single-cell cohort, we further examined a bulk transcriptomic dataset of leukemic stem cell fractions. Building upon the study by Stanley W. K. Ng et al., 83 samples from 78 AML patients were separated into 227 fractions according to CD34 and CD38 expression, and leukemic stem cell (LSC) activity was subsequently determined by xenotransplantation into NSG mice, yielding 138 LSC + and 89 LSC− fractions. When applying our m7G risk model to these 227 fractions, the LSC+ group exhibited significantly higher m7G risk scores than the LSC− group (*p* < 0.001). Moreover, the m7G risk score showed moderate discriminatory performance for identifying LSC+ fractions, with a mean AUC of 0.639 across 1,000 bootstrap resamples (Fig. 2F).

Further analyses of the m7G regulatory genes between LSC + and LSC– phenotypes highlighted that METTL1 and TRMT112 were significantly upregulated in the LSC+ group, whereas QKI and RAB27A were distinctly overexpressed in the LSC– group (Fig. 2G). Ng et al. constructed an LSC17 signature comprising 17 differentially expressed genes upregulated in the LSC+ group, which served as a marker of high leukemic stemness risk in AML. Utilizing the LSC17 signature, we calculated the high stemness risk scores for AML samples within the beat and TCGA arrays, noting a significant upregulation of LSC17 risk scores within the m7G high-risk group (*p* < 0.001, Supplementary Fig. 5A-B). Interestingly, the LSC17 signature genes, including SOCS2, CD34, and NYNRIN, were significantly overexpressed in the high-risk group, consistently observed across the GSE37642, BEAT, and TCGA cohorts (Supplementary Fig. 5C). Further analysis of SOCS2 in relation to AML prognosis in these three datasets identified it as a poor prognostic marker. In contrast, CD34 and NYNRIN showed no significant correlation with AML prognosis (Supplementary Fig. 5D).

### Higher m7G risk score indicates poorer drug treatment response

**Fig. 3:**
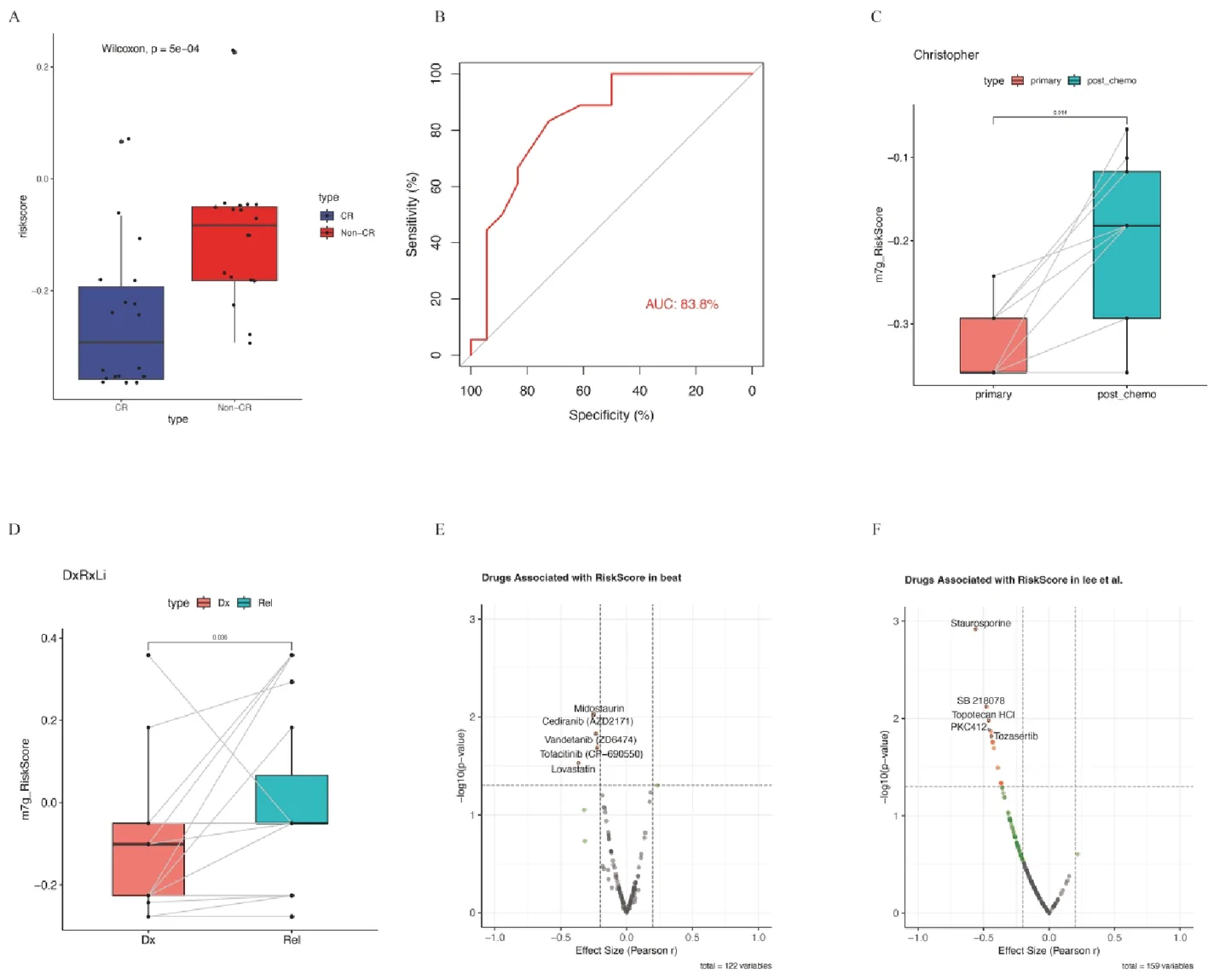
Identifying drugs with higher sensitivity in the high-risk group of the m7G risk model. **A** The box plot compares the distribution of m7G risk scores between the treatment outcomes of complete remission and induction failure groups. The risk scores of AML samples in the induction failure group are significantly elevated. The ROC curve evaluates the ability of the risk scores to predict treatment responses, as shown in **B**. **C**–**D** Using data from diagnosis-relapse paired AML samples in the studies by Christopher and Li, we compared the changes in m7G risk scores between diagnosis and relapse. The results indicate that the risk scores are significantly elevated in the relapse group. **E**–**F** Using drug sensitivity data from AML samples in the studies by Beat and Lee et al., we analyzed the correlation between drug IC50 values and risk scores. Volcano plots were generated based on p-values and correlation coefficients, highlighting the top five drugs with the strongest negative correlations. A p-value of < 0.05 indicates statistical significance

In previous analyses, the m7G risk model was identified as an independent prognostic factor. We aimed to determine whether the m7G risk model could predict drug treatment responses in AML patients. The GSE103424 dataset includes 36 AML samples, comprising 18 induction failure samples and 19 complete remission samples. We calculated the m7G risk scores for all samples. Comparing the risk score distributions between the two groups, we found that the risk scores in the induction failure group were significantly higher than those in the complete remission group (Fig. 3A). Furthermore, ROC analysis was conducted to assess the discriminatory power of the risk scores for treatment response, yielding an AUC value of 83.8% (Fig. 3B). These findings suggest that the m7G risk model can reliably predict treatment responses, which may partly explain its accuracy in forecasting AML prognosis.

### m7G risk scores significantly increase in relapsed AML patients

Relapse following chemotherapy remains a significant challenge in AML treatment. We sought to investigate changes in m7G risk scores in relapsed AML samples. Christopher et al. and Li et al. provided transcriptome data for paired diagnosis-relapse AML samples in their studies. We calculated the m7G risk scores for both initial diagnosis and relapse samples and analyzed the changes between the two groups using paired Wilcoxon tests. The results demonstrated a significant increase in m7G risk scores in the relapse group (Fig. 3C-D). These findings suggest that the m7G risk score reflects chemotherapy-associated shifts in the AML transcriptomic state, which may arise from changes in clonal composition (e.g., expansion of specific AML clones).

### Identifying drugs sensitive to high-risk groups

The high m7G risk group is associated with poorer prognosis and reduced response to chemotherapy. Consequently, we aimed to identify drugs that are more effective in high-risk AML samples using ex vivo drug sensitivity data. The BEAT AML dataset provides ex vivo drug sensitivity data for 122 small molecule inhibitors across AML patient samples. By analyzing the correlation between the IC50 values of these drugs and the m7G risk scores in AML samples, and applying the criteria of *p* < 0.05 and a correlation coefficient less than − 0.2, we identified five drugs (Vandetanib [ZD6474], Tofacitinib [CP-690550], Midostaurin, Cediranib [AZD2171], Lovastatin) whose IC50 values were significantly negatively correlated with the m7G risk scores (Fig. 3E). Among these, Vandetanib (ZD6474), Midostaurin, and Cediranib (AZD2171) target tyrosine kinases, while Tofacitinib (CP-690550) targets JAK. We further analyzed another dataset from Lee et al., which includes transcriptomic data from 30 AML samples and IC50 values for 159 chemotherapy drugs. Using the same method to analyze the correlation between drug sensitivity and risk scores, we found that 11 drugs exhibited higher sensitivity in the high-risk group. The top five drugs with the strongest correlation were Staurosporine, SB 218,078, Topotecan HCl, PKC412, and Tozasertib (Fig. 3F). By integrating data from both datasets, we identified several drugs that demonstrate better efficacy in the high m7G risk group.

## Discussion

In this study, we characterized AML heterogeneity using an m7G regulator–associated transcriptional framework. By clustering TCGA-LAML samples based on the expression of m7G regulatory genes, we identified two distinct m7G-related phenotypes with different survival outcomes and cytogenetic/hierarchy features. Building on the transcriptional differences between these phenotypes, we developed a cross-platform gene-pair–based m7G risk score and demonstrated its robust prognostic value across internal and external cohorts. Beyond survival prediction, the m7G risk score was associated with treatment response, relapse dynamics, stemness-related properties, and drug sensitivity patterns, supporting its potential utility for refined risk stratification in AML.

A key methodological contribution of our work is the use of a gene-pair strategy to construct the m7G signature. The final model comprises five gene pairs (ETS2|AGTPBP1, LY6E|AGTPBP1, PNMA1|DGKG, ACOT7|STAR, and IL2RA|TCF4). Compared with models relying on absolute expression levels, gene-pair scoring is less sensitive to cross-platform variation and batch effects, improving portability between RNA-seq and microarray datasets [37]. Biologically, the selected pairs may reflect coordinated programs relevant to AML progression. For example, ETS2 has been reported to be amplified and overexpressed in AML subsets with chromosome 21 abnormalities and complex karyotypes, and may influence proliferation and differentiation programs [38]. DGK family activity has also been implicated in AML cell viability and cell-cycle regulation, supporting the relevance of DGKG-associated states [39]. Moreover, IL2RA and TCF4 represent immune signaling and transcriptional circuitry with potential relevance to AML biology, and the IL2RA|TCF4 pair carried the largest coefficient in our model, suggesting that this axis may capture a survival-associated state [40]. Notably, prior work indicates that TCF4-encoded ITF2B can repress IL2RA transcription via a negative regulatory element, consistent with an antagonistic relationship between these genes. While these observations provide biological plausibility, the gene-pair signature should primarily be interpreted as a predictive surrogate of risk-associated transcriptional states rather than a direct mechanistic map.

Clinically, patients in the high-risk group defined by the m7G risk score showed significantly shorter overall survival across cohorts. Furthermore, the score discriminated induction outcomes, with higher scores observed in patients with chemotherapy induction failure, supporting a link between the high-risk state and primary treatment resistance. In addition, m7G risk scores were significantly increased in diagnosis–relapse paired samples, suggesting that this transcriptional risk program may become more prominent after therapy and may track disease evolution under treatment pressure. Together, these findings support the clinical relevance of the m7G risk score not only for baseline prognostication but also for capturing treatment-associated dynamics.

A central biological observation is the close relationship between the m7G risk score and leukemic stemness, a major determinant of AML persistence and relapse. Leukemic stem cells can evade the cytotoxic effects of AML treatment, and variations in leukemic stemness contribute to heterogeneous treatment responses [4, 5]. In our analyses, high-risk samples were enriched for primitive hierarchy states in bulk cohorts, and single-cell analysis further supported an association between high risk and a more stem-like cellular composition, with enrichment of HSC/MPP-like populations in the high-risk group. Consistently, in a transcriptomic dataset of functionally defined LSC fractions, LSC+ fractions exhibited significantly higher m7G risk scores than LSC− fractions, indicating that the score captures features associated with functional stemness [6]. These observations align with prior evidence that m7G regulators can remodel RNA metabolism and translation programs relevant to malignant states [11, 21]. We also observed that SOCS2 was elevated in the high-risk group and enriched in LSC+ fractions. Although SOCS2 has been reported to be regulated by RNA modification pathways such as m6A in other malignancies, whether SOCS2 is directly regulated by m7G-associated mechanisms in AML remains to be determined [41]. Therefore, SOCS2 should currently be viewed as a stemness-associated marker correlated with the m7G high-risk state, rather than evidence of direct m7G-mediated regulation. Notably, QKI was more highly expressed in mature hierarchy states and RAB27A was reduced in primitive states in our bulk hierarchy analyses (Supplementary Fig. 4), and higher RAB27A expression was associated with better overall survival in The Cancer Genome Atlas (TCGA) (Supplementary Fig. 2). Consistently, in the functionally defined LSC fraction dataset, QKI and RAB27A were distinctly overexpressed in the LSC− fraction (Fig. 2G), suggesting that these regulators may preferentially reflect a more differentiated/non-LSC transcriptional state rather than functional leukemic stemness per se. From an m7G perspective, QKI has been reported to act as an internal m7G-binding RNA-binding protein that interacts with the stress-granule core protein G3BP1 and shuttles internal m7G-modified transcripts into stress granules, thereby modulating mRNA stability and translation under stress [42]. This mechanism provides biological plausibility that higher QKI in LSC− fractions may be linked to post-transcriptional stress-adaptation programs. In parallel, QKI has also been implicated in myeloid differentiation circuitry (e.g., a C/EBPα–QKI5 feedback axis in committed macrophage progenitors), consistent with a differentiation-associated expression pattern [43]. For RAB27A, its established role in regulated exocytosis/granule release in neutrophils supports an association with mature myeloid secretory functions rather than stem-like programs [44]. Moreover, Rab27a-dependent exosome secretion has been shown to contribute to AML niche remodeling, and disrupting Rab27a-mediated exosome release can delay leukemia development in vivo, suggesting that higher RAB27A in LSC− fractions may also reflect vesicle-mediated microenvironmental communication [45]. Collectively, these observations highlight that m7G regulators can exhibit stemness-fraction–specific expression patterns, potentially capturing differentiation- and stress/secretory-state heterogeneity within AML.

We further explored therapeutic implications by integrating ex vivo drug sensitivity datasets and identified several compounds whose IC50 values were negatively correlated with the m7G risk score, suggesting preferential activity in high-risk AML. These results are best interpreted as hypothesis-generating candidates for risk-adapted therapeutic prioritization. For instance, topotecan (a TOP1 inhibitor) emerged among sensitivity-associated drugs in our analyses and has been evaluated in combination regimens for relapsed/refractory AML, indicating that TOP1-targeted strategies may merit further evaluation in high-risk strata. In addition, prior work has supported the rationale of TOP1-targeted strategies in specific genetic contexts in hematologic malignancies [46]. However, because ex vivo sensitivity does not directly translate into clinical efficacy, and because regimen response depends on multiple clinical factors, prospective validation and functional testing in model systems stratified by m7G risk will be necessary before clinical translation.

This study has several limitations. First, although our framework is motivated by m7G biology, we inferred m7G-related relevance primarily from the expression patterns of m7G regulators and downstream transcriptional programs rather than directly quantifying m7G modification levels. Future studies incorporating direct measurements (e.g., LC–MS/MS quantification of m7G nucleosides and/or m7G profiling methods such as m7G-RIP/MeRIP-based sequencing) in relevant cellular compartments (e.g., LSC-enriched versus bulk blast populations) will be critical to establish mechanistic links. Second, integrating multiple cohorts required restricting analyses to genes shared across platforms, which may have excluded informative genes not consistently measured. Third, immune- and differentiation-related findings derived from bulk transcriptomic inference and single-cell annotation should be interpreted as associative, and additional orthogonal validation will strengthen these conclusions. Finally, the drug sensitivity associations require experimental validation in independent cohorts and functional models.

In summary, our study delineates AML heterogeneity through an m7G regulator–associated transcriptional lens, provides a robust and portable gene-pair risk score with consistent prognostic performance, and links the high-risk state to adverse cytogenetics, treatment resistance, relapse dynamics, and stemness-related features. These findings support the m7G risk score as a practical tool for refined risk assessment and a framework for generating testable hypotheses for risk-adapted therapy in AML.

## Supplementary Information

Below is the link to the electronic supplementary material.

Supplementary Material 1.

Supplementary Material 2. Fig. 1. Workflow for deriving m7G risk scores.

Supplementary Material 3. Fig. 2. SNP and CNV Distribution and Their Prognostic Relevance to m7g Regulatory Genes in AML. (A) SNP profiling of QKI. (B) Incidence rate of CNVs in the 7 m7g regulatory genes within the TCGA LAML dataset. (C) Correlational analysis between CNV events and gene expression levels. (D) Overall survival disparities between groups with WDR4 CNVs versus wild-type WDR4. (E) Survival comparison between RNMT CNV carriers and wild-type counterparts. (F-K) Stratification into high and low expression cohorts based on the 7 m7g regulatory genes’ expression, followed by an analysis of their overall survival differences.

Supplementary Material 4. Fig. 3. SNP and CNV Distribution and Their Prognostic Relevance to m7g Regulatory Genes in AML. (A) The LASSO regression method was employed to devise a penalty function, leading to a refined prognostic model; this is evidenced by the attenuation of coefficients for minor variables to zero with increasing log lambda values, and a concurrent diminution in the partial likelihood bias. (B) A bubble chart exhibits 11 KEGG pathways concurrently enriched in upregulated genes within both Cluster A and the high-risk m7g group. Solid bubbles signify the high-risk cohort, whereas hollow ones denote Cluster A. (C) shows a boxplot comparing the proportions of CD8 + effector T cells between the high- and low-m7G-risk groups, based on analyses of a single-cell RNA-seq dataset.

Supplementary Material 5. Fig 4. Expression Patterns of the 6 m7g Regulatory Genes Among Diverse Cellular Hierarchy Subgroups. (A-B) Distributions within TCGA and BEAT datasets respectively.

Supplementary Material 6. Fig 5. m7g Risk Score’s Significant Association with AML Stemness. (A-B) LSC-17 risk scores exhibit pronounced overexpression in the m7g high-risk group across both BEAT and TCGA datasets. (C) The expression of SOCS2, CD34, and NYNRIN is significantly elevated in the high-risk groups within the TCGA, BEAT, and GSE37642 datasets. (D) The forest plot illustrates the results of univariate Cox regression analysis for the expression of SOCS2, CD34, and NYNRIN across the three datasets (TCGA, BEAT, and GSE37642).

## Data Availability

No new data were generated in this study. The data analyzed in this study are publicly available from databases, as described in the Methods section. Code used in the analysis is available upon reasonable request by contacting the corresponding author.
